# Using the Kulldorff’s scan statistical analysis to detect spatio-temporal clusters of tuberculosis in Qinghai Province, China, 2009–2016

**DOI:** 10.1186/s12879-017-2643-y

**Published:** 2017-08-21

**Authors:** Huaxiang Rao, Xinyu Shi, Xi Zhang

**Affiliations:** 1 Institute for Communicable Disease Control and Prevention, Qinghai Center for Disease Control and Prevention, No.55 Bayi middle Road, Xining, Qinghai 810007 China; 2 Operational Department, The Second Hospital Affiliated to Shanxi Medical University, Taiyuan, Shanxi 030001 China; 3 Clinical Research Center, Xinhua Hospital Affiliated to Shanghai Jiao Tong University School of Medicine, No. 1665 Kongjiang Road, Shanghai, 200092 China

**Keywords:** Tuberculosis, SaTScan, Space-time clustering, Qinghai

## Abstract

**Background:**

Although the incidence of tuberculosis (TB) in most parts of China are well under control now, in less developed areas such as Qinghai, TB still remains a major public health problem. This study aims to reveal the spatio-temporal patterns of TB in the Qinghai province, which could be helpful in the planning and implementing key preventative measures.

**Methods:**

We extracted data of reported TB cases in the Qinghai province from the China Information System for Disease Control and Prevention (CISDCP) during January 2009 to December 2016. The Kulldorff’s retrospective space-time scan statistics, calculated by using the discrete Poisson probability model, was used to identify the temporal, spatial, and spatio-temporal clusters of TB at the county level in Qinghai.

**Results:**

A total of 48,274 TB cases were reported from 2009 to 2016 in Qinghai. Results of the Kulldorff’s scan revealed that the TB cases in Qinghai were significantly clustered in spatial, temporal, and spatio-temporal distribution. The most likely spatio-temporal cluster (*LLR* = 2547.64, *RR* = 4.21, *P* < 0.001) was mainly concentrated in the southwest of Qinghai, covering seven counties and clustered in the time frame from September 2014 to December 2016.

**Conclusion:**

This study identified eight significant space-time clusters of TB in Qinghai from 2009 to 2016, which could be helpful in prioritizing resource assignment in high-risk areas for TB control and elimination in the future.

**Electronic supplementary material:**

The online version of this article (doi:10.1186/s12879-017-2643-y) contains supplementary material, which is available to authorized users.

## Background

Tuberculosis (TB) is an infectious disease caused by *Mycobacterium tuberculosis*. Over 80% of the new TB cases, globally, were reported in developing countries. According to a World Health Organization report, the TB burden of China is the second largest in the world [1]. In recent years, although the Chinese Government has paid an increasing amount of attention to the control of TB, prevention measures are still insufficient, especially in areas with inadequate medical resources, such as Qinghai, a province where most of the population suffers a high risk of TB even now [2].

A large number of studies on the spatial and temporal distribution of TB have demonstrated that TB has a highly complex dynamics and is spatially heterogeneous at the provincial, national, and international levels during certain periods of time; however, the variations in small area are always be ignored by using a relatively large scale [3–6]. In our previous study, the Moran’s *I* spatial autocorrelation analysis method was used to analyze the TB incidence data from 2009 to 2013 in Qinghai and found that the distribution of TB in this province was not random [7]. However the Global Moran’s *I* spatial autocorrelation analysis only evaluates the distribution characteristics of the disease in several specific time points. Moreover, this method can not estimate the risk level of high-risk cluster areas [8–11]. It is a known fact that time is a critical confounder that might directly bias the determination of the high-risk regions of TB. The Kulldorff’s space-time scan statistical method can detect the distribution characteristics in both the temporal and spatial axes, bringing them closer to real-world conditions [12–15]. Additionally, the relative risk of disease in a cluster area can be estimated by comparing it with the area outside the cluster area. This method has been used wildly in the epidemiology studies of infectious diseases [16–19].

Analyzing and evaluating the spatio-temporal patterns and trends of TB in Qinghai is necessary for TB control and elimination. In this study, our aim was to use the Kulldorff’s scan statistical analysis to explore the spatial, temporal, and space-time dynamics of TB at the county level in Qinghai.

## Methods

**Fig. 1:**
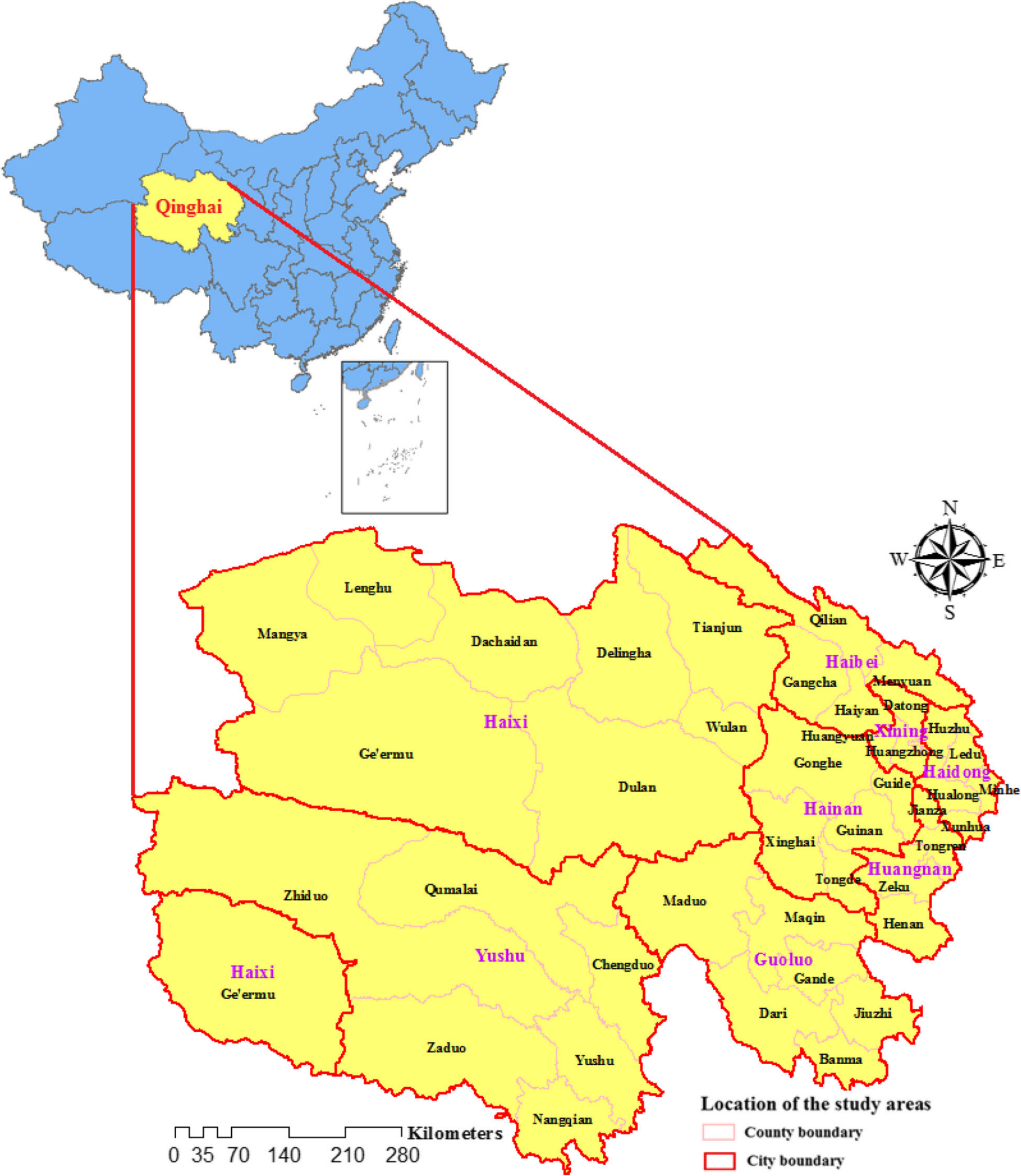
Location of Qinghai province in China. The statistical map was created by using ArcGIS software (version 10.2.2, ESRI Inc., Redlands, CA, USA)

Qinghai is located in the northwest China and lies to the northeast of the Qinghai-Tibet Plateau. The average altitude is 3000–5000 m. Qinghai comprises eight prefectures, including a total of 46 counties (Fig. 1). The province is comparatively less developed, with a high annual incidence of TB. The total population is about 5.7 million people.

### TB data

We collected data (2009–2016) of TB cases in Qinghai from the China Information System for Disease Control and Prevention (CISDCP). We also extracted the demographic data of 46 counties from Qinghai’s statistical yearbooks (2010–2017). TB is one of the notifiable infectious diseases in China. It is mandated that each case of TB must be reported online within 24 h after diagnosis in a hospital. Cases of TB were diagnosed using radiography, pathogen detection, and pathologic diagnosis, based on the diagnostic criteria recommended by the National Health and Family Planning Commission of the People’s Republic of China (2008).

A total of 48,274 incident cases of TB and 84 TB-related deaths were reported across 771 hospitals and medical institutions in Qinghai from January 2009 to December 2016. In this study, 48,165 cases, aggregated at the county level monthly, were analyzed to detect the spatio-temporal high-risk areas of TB. And 109 cases without detailed information on the residential address were excluded from the analysis. In order to check the missing reports of TB, we randomly selected 261 from all 771 hospitals and medical institutions, and double checked all the medical records of these selected institutions. No missing cases or outbreaks of TB were recorded.

### Statistical methods

We used Kulldorff’s space-time scan statistical analysis to detect the temporal, spatial, and space-time clusters of TB, and to verify whether the geographic clustering of TB was caused by random variation or not [20]. Since the population in several areas was very small, we used the radius of the population coverage instead of the geographical radius. The discrete Poisson probability model was used for scanning since the TB incidence was not very high [18]. The window with the maximum likelihood is defined as the most likely cluster area, and other clusters with statistically significant log-likelihood ratios (*LLR*) were defined as the secondary potential clusters. The *P*-values of *LLR* were estimated through 9999 Monte Carlo simulations [16, 18]. A *P*-value <0.05 indicates a significantly high risk inside of the scan window, which might be a potential cluster of a high risk of TB. The relative risk (*RR*) of TB in each cluster was calculated to evaluate the risk of TB in the cluster areas [12, 21, 22].

The results of spatio-temporal scan are sensitive to various parameters, like the maximum cluster sizes of spatial and temporal. Thus, the selection of the maximum radius of the spatial scanning window and the maximum length of the temporal scanning window were very important [23]. In order to select optimal parameters, we analyzed the data of 2009 using the maximum spatial cluster sizes from 4% to 50% of total population at risk by increments of 1%. The radius was considered as an optimal radius for analysis if there were fewer overlaps between the areas defined by the radius, and the biggest area covered less than seven counties or 15% of all the counties [24, 25]. Similarly, we found an optimal temporal cluster size by testing the maximum temporal cluster sizes from 10% to 50% of the total study period by increments of 1% to analyze the data of the preceding 5 years (2009–2013). Based on the the optimal spatio-temporal parameters, retrospective space-time scanning analysis was applied to identify the geographic areas and time periods of potential clusters with significantly higher TB incidents than that of nearby areas.

We also used Global Moran’s *I* spatial autocorrelation analysis to depict the spatial clustering of annual TB incidence at the county level. The Moran’s *I* > 0, = 0, and <0 indicate a positive spatial autocorrelation, random distribution, and negative spatial autocorrelation, respectively [12].

Additionally, we conducted time series seasonal decomposition analysis to identify the seasonality of TB incidence in Qinghai province [26–28]. The seasonal index was also calculated to examine the seasonal pattern of TB. The index was calculated as the ratio of the average number of cases for a given month to the average monthly incidents of 8*12 months (2009–2016). An index value close to 1.0 indicates no seasonal trend [29].

The SaTScan™ software (v 9.4.1, Kulldorff and Information Management Services, Inc.) was used for spatial, temporal, and spatio-temporal analyses. Then, we used ArcGIS (v10.2.2, ESRI Inc., Redlands, CA, USA) to visualize the relative risk of TB in high-risk cluster areas. Open GeoDa software (Arizona State University, AZ, USA) was used for Global Moran’s *I* spatial autocorrelation analysis. *P* < 0.05 indicates a statistical significance.

## Results

### Determination of the optimal time window for temporal scanning

We conducted temporal scanning by using the time window with the length that covers 10–50% of the total study period, by increments of 1%. The scanning results indicated that the high-risk cluster of TB was predominantly concentrated in the time period between January 2012 and May 2013 (Additional file 1: Table S1). Therefore, the maximum temporal cluster size was set as 30% in this study. For each year, the maximum scan time length was 3 months.

### Determination of the optimal space window for spatial scanning

**Fig. 2:**
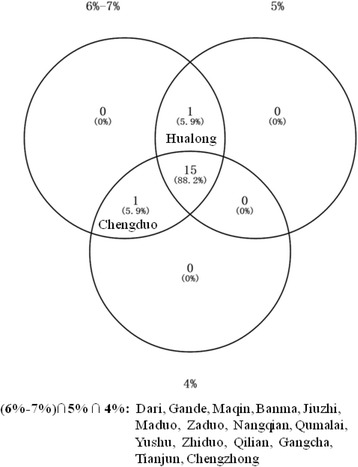
Venn diagram of spatial clustering for TB incidents in Qinghai, China, 2009. The scan window used in this analysis was set to be 7% of population

In order to detect an appropriate space window for the spatial scan, we conducted several times of spatial scanning using different maximum circular spatial windows. We started from the radius covering from 4% to 50% of the population, increasing by 1% each time. The results are shown in Additional file 1: Fig. S1. When the maximum spatial scan size was set to 8–50%, the high-risk clustering areas overlapped, and the most likely cluster covered more than 15% of all the counties. While for the sizes of 4–7%, selfsame area was detected as the most likely cluster area, but the secondary clusters were slightly different. The cluster areas identified using the windows of 6–7% covered the largest high-incidence areas. According to the Venn diagram (Fig. 2), we finally set 7% as the maximum circular spatial window for spatial scanning, covering a population of 0.392 million. Two counties, Huangzhong and Datong, were not included in the scanning window. The incidence rates of TB in these two counties were relatively low. Therefore, the exclusion would not be influential.

### Distribution of TB temporal clustering

**Fig. 3:**
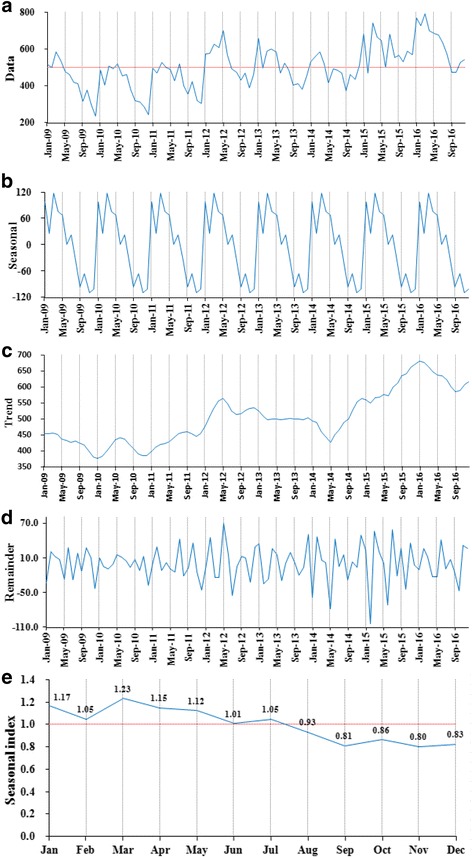
The seasonal distribution of monthly TB incidents, between years of 2009 and 2016, in Qinghai, China. **a**: Time-series of monthly TB incidents; **b**: A seasonal trend was decomposed from the time-series of TB incidents; **c**: A long-term trend was decomposed from the time-series of TB incidents; **d**: The residual data after excluding of seasonality and a long-term trend; **e**: Estimated seasonal index of 12 months ranged from 0.80 to 1.23, and the maximum value was recorded in March

The time series seasonal decomposition analysis of TB incidents showed a significant seasonal periodicity, but the seasonal trend was not obvious between 2014 and 2015 (Fig. 3a and b). This was consistent with the result of the seasonal index (Fig. 3e). The maximum seasonal index value was 1.23 in March, and the value appeared to be less than 1.0 after July. There was a slowly increase trend for TB incidents from 2009 to 2016 (Fig. 3c). The temporal cluster analysis also showed that TB incidents were mainly concentrated in the spring and early summer, annually, ranged from January to May. The high aggregated period for TB was observed in all districts from January 2015 to August 2016 (*LLR* = 353.28, *P* < 0.05). During this period, a total of 12,746 TB cases were reported, and the risk of TB related incidents was 32% (*RR* = 1.32) higher than that in other time periods (Table 1).

**Table 1:** Temporal clustering of TB incidents in Qinghai, China, 2009–2016

Year	Cluster time frame	Observed cases	Expected cases	*RR*	*LLR*	*P*-value
2009	2009/2/1 to 2009/4/30	1616	1246.49	1.43	68.29	0.001
2010	2010/3/1 to 2010/5/31	1514	1220.95	1.35	44.83	0.001
2011	2011/2/1 to 2011/4/30	1490	1270.14	1.24	24.27	0.001
2012	2012/3/1 to 2012/5/31	1928	1593.41	1.30	44.98	0.001
2013	2013/1/1 to 2013/3/31	1743	1492.52	1.24	26.94	0.001
2014	2014/2/1 to 2014/4/30	1665	1424.98	1.24	25.81	0.001
2015	2015/3/1 to 2015/5/31	2051	1812.53	1.18	20.41	0.001
2016	2016/1/1 to 2016/3/31	2286	1882.90	1.31	54.99	0.001
2009–2016	2015/1/1 to 2016/8/31	12,746	10,291.14	1.32	353.28	0.001

### Distribution of TB spatial clustering

Spatial clustering analysis of the entire 8 years identified a total of nine statistically significant high-risk areas, covering a total of 21 counties. Similarly, the Global Moran’s *I* values of each year at the county level also indicated a positive spatial autocorrelation in Qinghai, ranging from 0.398 to 0.631 (all *P* < 0.05). The high-risk areas with a relative risk greater than three, including the most likely cluster area and two secondary cluster areas, were mainly concentrated in the southwest of Qinghai. The center of the most likely cluster area was located in Dari County, 33.48° N and 99.41° E (*LLR* = 3225.58, *P* < 0.001). This circular area covered six counties with a radius of 184.91 km, including Dari, Gande, Maqin, Banma, Jiuzhi, and Maduo. The total number of TB cases was 5408, and the risk of TB related incidents was 2.97 times (*RR* = 3.97) higher than that outside this area (Table 2 and Additional file 1: Fig. S2). The incidences of TB inside the cluster areas were significantly higher than that in the areas outside every year (Table 3).

**Table 2:** Spatial clustering of TB incidents in Qinghai, China, 2009–2016

Cluster type	Coordinates/Radius	N	Cluster counties	Observed cases	Expected cases	*RR*	*LLR*	*P*-value
Most likely cluster	(33.48 N, 99.41 E)/184.91 km	6	Dari, Gande, Maqin, Banma, Jiuzhi, Maduo	5408	1488.94	3.97	3225.58	<0.001
Secondary cluster 1	(34.86 N, 95.13 E)/229.73 km	4	Qumalai, Chengduo, Zaduo, Zhiduo	5244	1410.00	4.05	3215.65	<0.001
Secondary cluster 2	(32.17 N, 96.13 E)/97.79 km	2	Yushu, Nangqian	4728	1674.66	3.02	1956.35	<0.001
Secondary cluster 3	(36.07 N, 102.23 E)/0 km	1	Hualong	2530	1838.12	1.40	121.59	<0.001
Secondary cluster 4	(35.51 N, 99.73 E)/107.12 km	3	Xinghai, Tongde, Guinan	2422	1815.02	1.35	95.75	<0.001
Secondary cluster 5	(35.43 N, 102.08 E)/0 km	1	Tongren	1106	780.65	1.43	61.08	<0.001
Secondary cluster 6	(38.32 N, 99.70 E)/96.82 km	2	Qilian, Gangcha	1063	768.15	1.39	51.40	<0.001
Secondary cluster 7	(37.46 N, 101.70 E)/0 km	1	Menyuan	1451	1291.45	1.13	9.75	<0.001
Secondary cluster 8	(35.90 N, 101.85 E)/0 km	1	Jianza	573	475.97	1.21	9.38	<0.001

**Table 3:** The characteristics of TB incidents in Qinghai, China, 2009–2016

Year	TB incidence inside the cluster (1/100000)						TB incidence outside the cluster (1/100000)						*u*	*P*-value
	*N*_1_	Minimum	*Q*_*L*_	Median	*Q*_*U*_	Maximum	*N*_2_	Minimum	*Q*_*L*_	Median	*Q*_*U*_	Maximum		
2009	17	129.80	154.28	215.01	265.32	623.13	29	0.00	58.52	76.36	90.52	121.62	5.61	<0.001
2010	15	110.72	160.23	251.38	328.18	524.69	31	3.68	48.88	71.12	92.51	208.15	5.21	<0.001
2011	16	103.18	127.91	203.88	298.01	679.25	30	14.71	51.89	71.77	95.04	194.73	5.10	<0.001
2012	15	148.88	174.96	355.84	431.45	773.49	31	14.46	57.62	83.45	124.70	432.02	4.96	<0.001
2013	21	113.83	149.79	273.93	407.32	696.89	25	14.46	45.49	70.34	87.53	117.72	5.77	<0.001
2014	18	135.87	148.03	281.35	421.50	582.58	28	16.12	47.09	82.27	99.61	140.12	5.63	<0.001
2015	18	142.62	202.73	372.60	641.43	844.60	28	3.45	42.35	77.28	126.45	163.62	5.63	<0.001
2016	16	194.60	314.00	413.62	739.70	1220.47	30	11.35	46.22	82.56	121.06	170.14	5.54	<0.001
2009–2016	21	117.67	141.39	263.79	412.80	736.42	25	12.92	49.21	69.03	95.87	116.00	5.79	<0.001

### Distribution of TB spatio-temporal clustering

**Fig. 4:**
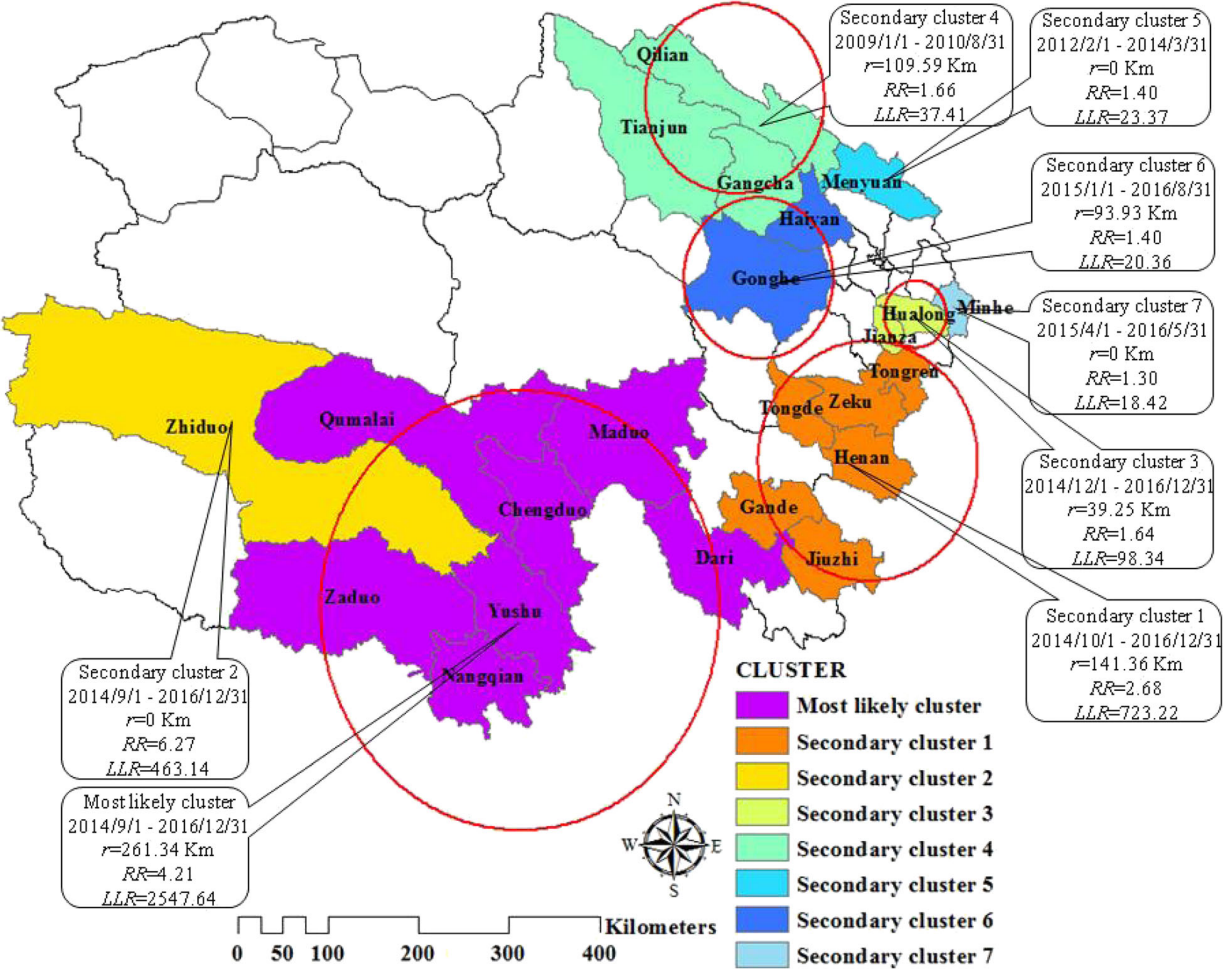
Spatio-temporal clustering of TB incidents at the county level in Qinghai, China, 2009–2016. A most likely cluster and seven secondary clusters were found by spatio-temporal scanning, which indicated an obvious trend of spatio-temporal clustering for TB incidents in Qinghai. The high-risk clusters were predominantly located in the southwest Qinghai between September 2014 and December 2016

The results of spatio-temporal cluster analysis suggested a special characteristic in temporal and spatial distribution for TB incidents in Qinghai. We detected a most likely cluster area and seven secondary cluster areas by using temporal and spatial scanning (Fig. 4). The most likely spatio-temporal cluster area was located at the southwest of the province, and the high-risk period was from September 2014 to December 2016 (*LLR* = 2547.64, *P* < 0.001). The center of this area was in Yushu County, 32.91° N and 96.68° E, which was a circular area with a radius of 261.34 km, covering seven counties: Yushu, Nangqian, Chengduo, Zaduo, Maduo, Qumalai, and Dari. A total of 3916 TB cases were reported in this area during the high-risk period, and the *RR* was 4.21 (Table 4).

**Table 4:** Spatio-temporal clustering of TB incidents in Qinghai, China, 2009–2016

Cluster type	Cluster time frame	Coordinates/Radius	N	Cluster counties	Observed cases	Expected cases	*RR*	*LLR*	*P*-value
Most likely cluster	2014/9/1 to 2016/12/31	(32.91 N, 96.68 E)/261.34 km	7	Yushu, Nangqian, Chengduo, Zaduo, Maduo, Qumalai, Dari	3916	991.33	4.21	2547.64	<0.001
Secondary cluster 1	2014/10/1 to 2016/12/31	(34.52 N, 101.57 E)/141.36 km	6	Henan, Zeku, Tongde, Tongren, Jiuzhi, Gande	2069	794.40	2.68	723.22	<0.001
Secondary cluster 2	2014/9/1 to 2016/12/31	(34.87 N, 92.61 E) / 0 km	1	Zhiduo	467	75.10	6.27	463.14	<0.001
Secondary cluster 3	2014/12/1 to 2016/12/31	(36.07 N, 102.23 E)/39.25 km	2	Hualong, Jianza	953	585.25	1.64	98.34	<0.001
Secondary cluster 4	2009/1/1 to 2010/8/31	(38.32 N, 99.70 E)/109.59 km	3	Qilian, Gangcha, Tianjun	346	209.32	1.66	37.41	<0.001
Secondary cluster 5	2012/2/1 to 2014/3/31	(37.46 N, 101.70 E)/0 km	1	Menyuan	471	338.24	1.40	23.37	<0.001
Secondary cluster 6	2015/1/1 to 2016/8/31	(36.45 N, 100.03 E)/93.93 km	2	Gonghe, Haiyan	405	290.12	1.40	20.36	<0.001
Secondary cluster 7	2015/4/1 to 2016/5/31	(36.15 N, 102.77 E)/0 km	1	Minhe	573	440.34	1.30	18.42	<0.001

## Discussion

In this study, spatial patterns and the secular trends of TB in Qinghai from 2009 to 2016 were examined using the Kulldorff’s scan statistical analysis. To the best of our knowledge, no other similar study has been done in this area. Our study demonstrated that there was a significantly space-time clustering in distribution of TB incidents in Qinghai province. The high-risk areas were mainly concentrated in the southwest Qinghai, and the temporal clusters were mainly concentrated in spring and early summer.

Kulldorff’s retrospective scan statistics take multiple testing problems into account, which is known as the most powerful method for evaluating geographical and temporal distribution by using routinely collected data [18]. This method has been used worldwide to detect the clusters of diseases [2, 4, 5, 30, 31]. As is known, in the temporal and spatial model, selection of a suitable time window and spatial window was very important for model identification. Currently, there are two methods for selecting the size of spatial window: one is based on the geographical area, and the other method is based on the population size covered by the scanning area [16]. In this study, we used the radius of population coverage, because deviation of population in different counties was very large. Similarly, size of time scanning window is another important parameter for analysis. Generally, the window size of time was set as 50% of the entire time period of the study. However, there exists some evidence with regard to whether or not this window size is reasonable [32]. Yue Ma et al. conducted a simulation study to explore how to choose an appropriate scanning window. They found that the window might be too large to include the low-risk area if the window covered 50% of the population [23]. Therefore, this situation might lead to a high false positive rate. However, the window which covered a smaller population might be too small to detect the real high-risk area, and the high-risk area would be separated. Thus, the high false negative rate would be an issue. Tango and Takahashi suggested that when using the irregular scan statistic to detect the aggregated region, the coverage area of a single region should not be more than 15% of the whole study area [24, 25]. In addition, several studies also suggested choosing an appropriate window which could identify the cluster areas with less overlap [4]. Based on the Tango’s criteria, we analyzed the data for many times by using one window value at a time. Finally, we selected the temporal window covering 30% of whole study period and the spatial window covering 7% of the population at risk. And the overlapping of the identified high-risk clusters was not observed.

Our temporal scanning results indicated that there was a high-risk period for TB incidents every year and during the entire 8 years, which mainly occurred in spring and early summer, from January to May. As is known, during winter, the reduction in exposure to ultraviolet rays from sunlight and the poor ventilation in indoor settings may increase the incidences of TB infections. Additionally, in the case of infectious diseases, time is needed for the symptoms to develop and patients may lack the knowledge on where to seek care for TB. All these factors may delay TB diagnosis and treatment [4]. Some studies reported that the average incubation period of TB infection ranges from 4 to 8 weeks, with a 2-month interval from the appearance of symptoms to medical diagnosis [33]. Therefore, the high-risk periods in spring and early summer complied with the disease characteristics. Such seasonal patterns were consistent with the previous studies done in Yunnan province, where is the registration peak of TB cases during spring [34].

Additionally, spatial scanning results displayed statistically significant 5–7 cluster areas for TB diseases in Qinghai each year, which were similar to the results of our previous study [7]. Compared with the median incidence rate of TB (69/100000) out of the cluster areas, TB incidence in our identified TB cluster was higher than 263/100000. This indirectly suggested a relatively high sensitivity of this scanning method. The spatio-temporal model used in this study simultaneously considered time and space distributions. Compared with the separated spatial scanning model and temporal scanning model, the time-space scanning makes a conclusion more closely to the real-world situation. Using this model to detect the spatio-temporal distribution of TB in Qinghai, from 2009 to 2016, we found that the high-risk counties were concentrated in the southwest Qinghai, from September 2014 to December 2016. During this period, the risk of TB infection in these areas was obviously higher than in other areas, especially in the Zhiduo County. In these areas, the inhabitants have very low income, as well as poorer living conditions and sanitation compared to the eastern region of Qinghai. Many studies have showed that poverty is one of the most important social factors responsible for the high prevalence of TB, and also the socio-economic status may contribute to the high risk of TB [4]. Our result indicated that further prevention and special TB control strategies should be considered in relation with the economical and sanitary level in the clustered areas.

Our study also demonstrated the usefulness of spatial and temporal clustering analysis using the ArcGIS and SaTScan to identify the significant space-time clusters of TB in Qinghai. This could be used to provide strategies for TB prevention at the county level. However, the study had limitations on analysis. First, it is important to note that the data were analyzed at the county level, which is not the smallest unit of administrative regionalization. Thus, we may exclude several critical factors. Second, the influence of weather and socio-economic factors were not included in this study.

## Conclusions

Our study analyzed the spatial, temporal, and space-time clusters of TB incidents at the county level in Qinghai, from 2009 to 2016, using the Kulldorff’s retrospective scan statistic methods. The spatial and temporal clusters were statistically significant every year, and the space-time scanning result indicated eight high-risk areas for TB incidents which were predominantly located in the southwest Qinghai. These results suggested that it is urgent to establish the preventive and controlling strategies to decrease the TB incidence in Qinghai by Qinghai government and the Center for Disease Control and Prevention.

## Additional file

Additional file 1:
**Table S1.** Temporal clustering of TB incidents monthly in Qinghai, China, 2009–2013. We set the maximum size for temporal scanning to be 18 months, nearly 30% of the total study period, by which the scan result was best to fit the raw time-series data of TB incidents. **Fig. S1.** Spatial clustering of TB incidents at the county level in Qinghai, China, 2009. **Fig. S2.** Spatial clustering of TB incidents at the county level in Qinghai, China, 2009–2016. (DOCX 682 kb)

## Abbreviations

CISDCP: China Information System for Disease Control and Prevention
GIS: Geographical information system
*LLR*: Log-likelihood ratio
*RR*: Relative risk
TB: Tuberculosis

## References

[1] Zumla A, George A, Sharma V, Herbert RH, Oxley A, Baroness Masham of I (2015). The WHO 2014 global tuberculosis report--further to go. Lancet Glob Health.

[2] Zhao F, Cheng S, He G, Huang F, Zhang H, Xu B (2013). Space-time clustering characteristics of tuberculosis in China, 2005-2011. PLoS One.

[3] Cao K, Yang K, Wang C, Guo J, Tao L, Liu Q, et al. Spatial-Temporal Epidemiology of Tuberculosis in Mainland China: An Analysis Based on Bayesian Theory. Int J Environ Res Public Health. 2016;13(5):e469. 10.3390/ijerph13050469 PMC4881094 27164117

[4] Ge E, Zhang X, Wang X, Wei X (2016). Spatial and temporal analysis of tuberculosis in Zhejiang Province, China, 2009-2012. Infect Dis Poverty..

[5] Areias C, Briz T, Nunes C (2015). Pulmonary tuberculosis space-time clustering and spatial variation in temporal trends in Portugal, 2000-2010: an updated analysis. Epidemiol Infect.

[6] Middelkoop K, Bekker LG, Morrow C, Zwane E, Wood R (2009). Childhood tuberculosis infection and disease: a spatial and temporal transmission analysis in a South African township. S Afr Med J.

[7] Rao HX, Zhang X, Zhao L, Yu J, Ren W, Zhang XL (2016). Spatial transmission and meteorological determinants of tuberculosis incidence in Qinghai Province, China: a spatial clustering panel analysis. Infect Dis Poverty..

[8] Sadeq M (2016). Spatial patterns and secular trends in human leishmaniasis incidence in Morocco between 2003 and 2013. Infect Dis Poverty..

[9] Varga C, Pearl DL, McEwen SA, Sargeant JM, Pollari F, Guerin MT (2015). Area-level global and local clustering of human Salmonella Enteritidis infection rates in the city of Toronto, Canada, 2007-2009. BMC Infect Dis.

[10] Lopez D, Gunasekaran M, Murugan BS, Kaur H, Abbas KM (2014). Spatial Big Data Analytics of Influenza Epidemic in Vellore. India Proc IEEE Int Conf Big Data.

[11] Zulu LC, Kalipeni E, Johannes E (2014). Analyzing spatial clustering and the spatiotemporal nature and trends of HIV/AIDS prevalence using GIS: the case of Malawi, 1994-2010. BMC Infect Dis.

[12] Xia J, Cai S, Zhang H, Lin W, Fan Y, Qiu J (2015). Spatial, temporal, and spatiotemporal analysis of malaria in Hubei Province, China from 2004-2011. Malar J.

[13] Vieira CP, Oliveira AM, Rodas LA, Dibo MR, Guirado MM, Chiaravalloti NF (2014). Temporal, spatial and spatiotemporal analysis of the occurrence of visceral leishmaniasis in humans in the City of Birigui, State of Sao Paulo, from 1999 to 2012. Rev Soc Bras Med Trop.

[14] Qian H, Huo D, Wang X, Jia L, Li X, Li J (2016). Detecting spatial-temporal cluster of hand foot and mouth disease in Beijing, China, 2009-2014. BMC Infect Dis.

[15] Kulldorff M, Nagarwalla N (1995). Spatial disease clusters: detection and inference. Stat Med.

[16] Abbas T, Younus M, Muhammad SA (2015). Spatial cluster analysis of human cases of Crimean Congo hemorrhagic fever reported in Pakistan. Infect Dis Poverty..

[17] Zhang Y, Shen Z, Ma C, Jiang C, Feng C, Shankar N (2015). Cluster of human infections with avian influenza A (H7N9) cases: a temporal and spatial analysis. Int J Environ Res Public Health.

[18] Alemu K, Worku A, Berhane Y (2013). Malaria infection has spatial, temporal, and spatiotemporal heterogeneity in unstable malaria transmission areas in northwest Ethiopia. PLoS One.

[19] Coleman M, Coleman M, Mabuza AM, Kok G, Coetzee M, Durrheim DN (2009). Using the SaTScan method to detect local malaria clusters for guiding malaria control programmes. Malar J.

[20] Jones SG, Kulldorff M (2012). Influence of spatial resolution on space-time disease cluster detection. PLoS One.

[21] Hjalmars U, Kulldorff M, Gustafsson G, Nagarwalla N (1996). Childhood leukaemia in Sweden: using GIS and a spatial scan statistic for cluster detection. Stat Med.

[22] Huang L, Kulldorff M, Gregorio D (2007). A spatial scan statistic for survival data. Biometrics.

[23] Ma Y, Yin F, Zhang T, Zhou XA, Li X (2016). Selection of the Maximum Spatial Cluster Size of the Spatial Scan Statistic by Using the Maximum Clustering Set-Proportion Statistic. PLoS One.

[24] Tango T, Takahashi K (2005). A flexibly shaped spatial scan statistic for detecting clusters. Int J Health Geogr.

[25] Tango T, Takahashi K (2012). A flexible spatial scan statistic with a restricted likelihood ratio for detecting disease clusters. Stat Med.

[26] Xu Z, Hu W, Zhang Y, Wang X, Zhou M, Su H (2015). Exploration of diarrhoea seasonality and its drivers in China. Sci Rep.

[27] Willis MD, Winston CA, Heilig CM, Cain KP, Walter ND, Mac Kenzie WR (2012). Seasonality of tuberculosis in the United States, 1993-2008. Clin Infect Dis.

[28] Xu Z, Hu W, Su H, Turner LR, Ye X, Wang J (2014). Extreme temperatures and paediatric emergency department admissions. J Epidemiol Community Health.

[29] Zhang Q, Lai S, Zheng C, Zhang H, Zhou S, Hu W (2014). The epidemiology of Plasmodium vivax and Plasmodium falciparum malaria in China, 2004-2012: from intensified control to elimination. Malar J.

[30] Wang WL, Wang HJ, Deng Y, Song T, Lan JM, Wu GZ (2016). Serological Study of An Imported Case of Middle East Respiratory Syndrome and His Close Contacts in China, 2015. Biomed Environ Sci.

[31] Zhang WY, Wang LY, Liu YX, Yin WW, Hu WB, Magalhaes RJ (2014). Spatiotemporal transmission dynamics of hemorrhagic fever with renal syndrome in China, 2005-2012. PLoS Negl Trop Dis.

[32] Wang LY, Zhang WY, Ding F, Hu WB, Soares Magalhaes RJ, Sun HL (2013). Spatiotemporal patterns of Japanese encephalitis in China, 2002-2010. PLoS Negl Trop Dis.

[33] Li XX, Wang LX, Zhang H, Du X, Jiang SW, Shen T (2013). Seasonal variations in notification of active tuberculosis cases in China, 2005-2012. PLoS One.

[34] Huang L, Li XX, Abe EM, Xu L, Ruan Y, Cao CL (2017). Spatial-temporal analysis of pulmonary tuberculosis in the northeast of the Yunnan province, People's Republic of China. Infect Dis Poverty.

