# Supplementary material for: Using the Kulldorff’s scan statistical analysis to detect spatio-temporal clusters of tuberculosis in Qinghai Province, China, 2009–2016
Source: BMC Infect Dis. 2017 Aug 21;17:578. doi: 10.1186/s12879-017-2643-y (PMC5563899; doi:10.1186/s12879-017-2643-y)
Supplement: Additional file 1: — Table S1. Temporal clustering of TB incidents monthly in Qinghai, China, 2009–2013. We set the maximum size for temporal scanning to be 18 months, nearly 30% of the total study period, by which the scan result was best to fit the raw time-series data of TB incidents. Fig. S1. Spatial clustering of TB incidents at the county level in Qinghai, China, 2009. Fig. S2. Spatial clustering of TB incidents at the county level in Qinghai, China, 2009–2016. (DOCX 682 kb) [file 12879_2017_2643_MOESM1_ESM.docx]

**Table S1 Temporal clustering of TB incidents monthly in Qinghai, China, 2009- 2013**

| Aggregation  length (%) | Cluster time  frame | Observed  cases | Expected  cases | *RR* | *LLR* | *P*-value |
| --- | --- | --- | --- | --- | --- | --- |
| 32-50 | 2012/1/1 to 2013/7/31 | 10257 | 8829.13 | 1.26 | 165.57 | 0.001 |
| 29-31 | 2012/1/1 to 2013/5/31 | 9265 | 7892.40 | 1.26 | 162.04 | 0.001 |
| 27-28 | 2012/2/1 to 2013/5/31 | 8694 | 7420.49 | 1.25 | 144.66 | 0.001 |
| 10-26 | 2012/1/1 to 2012/6/30 | 3642 | 2770.58 | 1.36 | 140.08 | 0.001 |

Note: We set the maximum size for temporal scanning to be 18 months, nearly 30% of the total study period, by which the scan result was best to fit the raw time-series data of TB incidents.

**Figure S1**

**
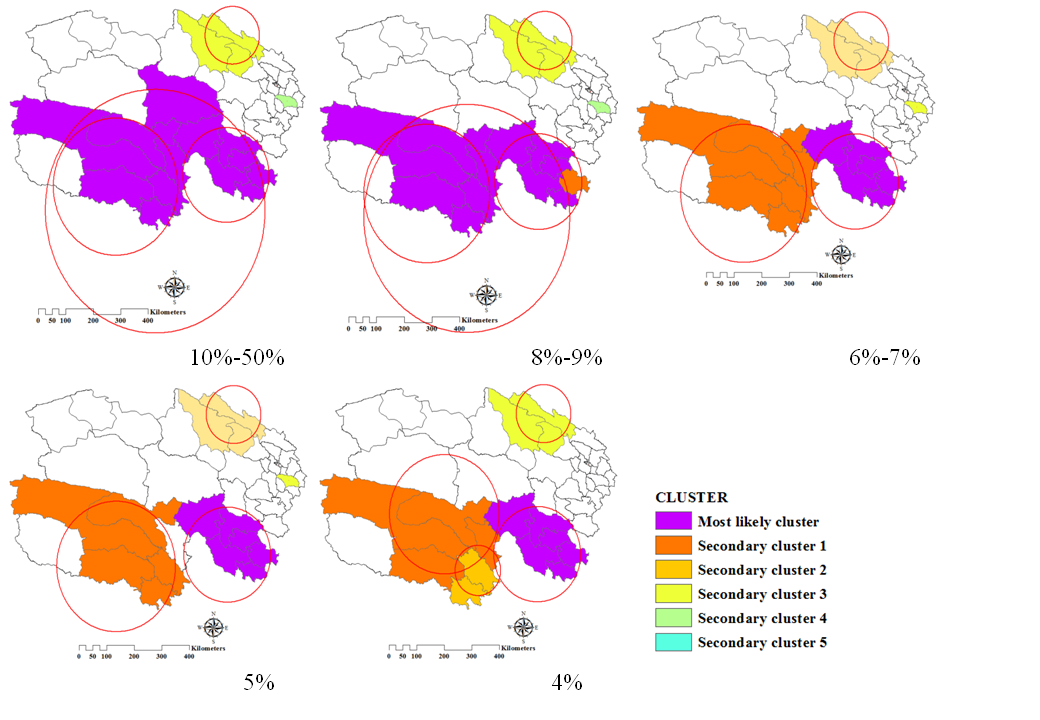
**

**Figure S2**

**
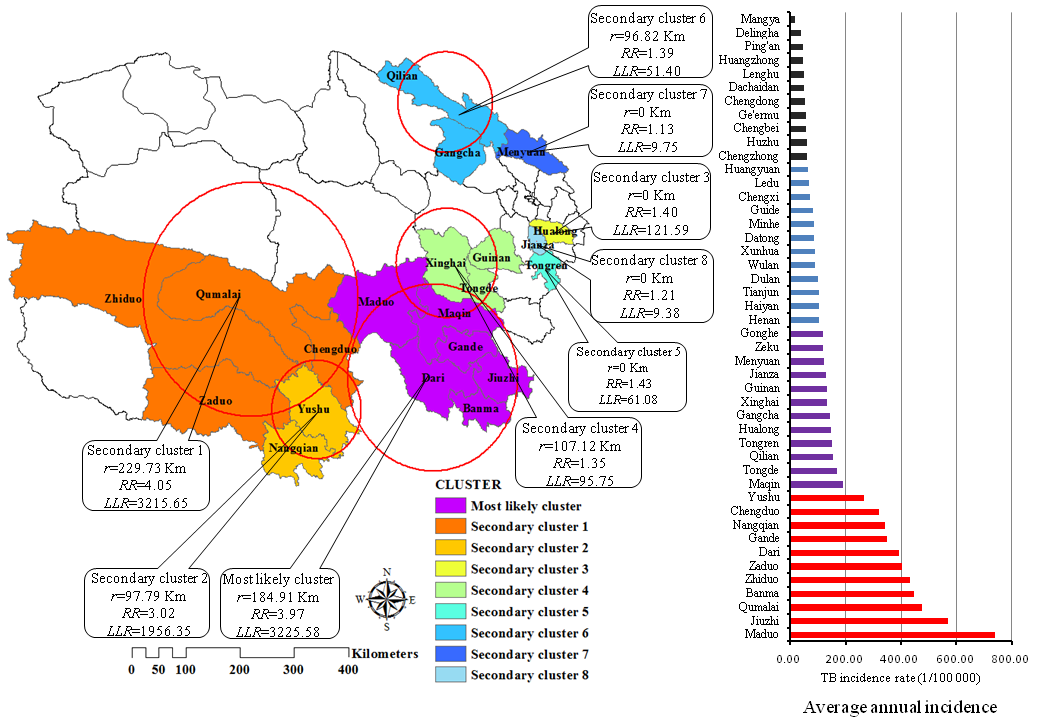
**
